# Hyaluronic Acid-Based Scaffolds as Potential Bioactive Wound Dressings

**DOI:** 10.3390/polym13132102

**Published:** 2021-06-26

**Authors:** Sibusiso Alven, Blessing A. Aderibigbe

**Affiliations:** Department of Chemistry, Alice Campus, University of Fort Hare, Alice 5700, Eastern Cape, South Africa; 201214199@ufh.ac.za

**Keywords:** wound dressings, bioactive agents, hyaluronic acid, wound management

## Abstract

The negative factors that result in delayed and prolonged wound healing process include microbial pathogens, excess wound exudates, underlying conditions, smoking, obesity, etc. Most of the currently used wound dressings demonstrate an inadequate capacity to treat wounds resulting from the factors mentioned above. The commonly used wound dressings include hydrogels, films, hydrocolloids, foams, fibers, sponges, dermal patches, bandages, etc. These wound dressings can be loaded with various types of bioactive agents (e.g., antibiotics, nanoparticles, anti-inflammatory drugs, etc.) to improve their therapeutic outcomes. Biopolymers offer interesting properties suitable for the design of wound dressings. This review article will be based on hyaluronic-acid-based scaffolds loaded with therapeutic agents for the treatment of wounds.

## 1. Introduction

Some known factors that contribute to chronic wounds include diabetes, prolonged bed rest, obesity, smoking, age, etc. A chronic wound is an injury that is not healed in a timely and orderly manner [1,2]. These wounds include leg ulcers, diabetic foot ulcers, etc. [2]. It is reported that the burden of treating chronic wounds contributes to an increase in healthcare costs worldwide. Approximately $20 billion is utilized every year to manage wounds in the United States of America [3]. Skin defects are a severe health threat because they expose the body to bacteria invasion, which can be fatal [4,5]. Wound healing is a complex and dynamic process of tissue formation. It occurs in four sequential stages: hemostasis phase, inflammatory phase; proliferation phase; and maturation phase, which is the final stage of wound healing [6,7].

There are several wound dressing materials that can be used for the treatment of wounds. Some of the dressings employed for wound healing include hydrogels [8,9,10,11,12,13,14,15], sponges [7,16], films [17,18,19,20], nanofibers [21,22], bandages [23,24,25,26,27], membranes [28,29,30,31,32,33,34], hydrocolloids [35], etc. These wound dressings are usually employed as the barrier against the infiltration of microbial pathogens into the wound environment. There is no single wound dressing that is suitable for wound care of all wound types. Wound dressings can be encapsulated with bioactive agents to enhance their therapeutic outcomes on wound healing. The bioactive agents that can be used in wound treatment include antibiotics, metal-based and polymer-based nanoparticles, anti-inflammatory agents, growth factors, etc. [36,37]. The wound dressings that are encapsulated with the agents mentioned above are called bioactive wound dressings.

**Figure 1:**
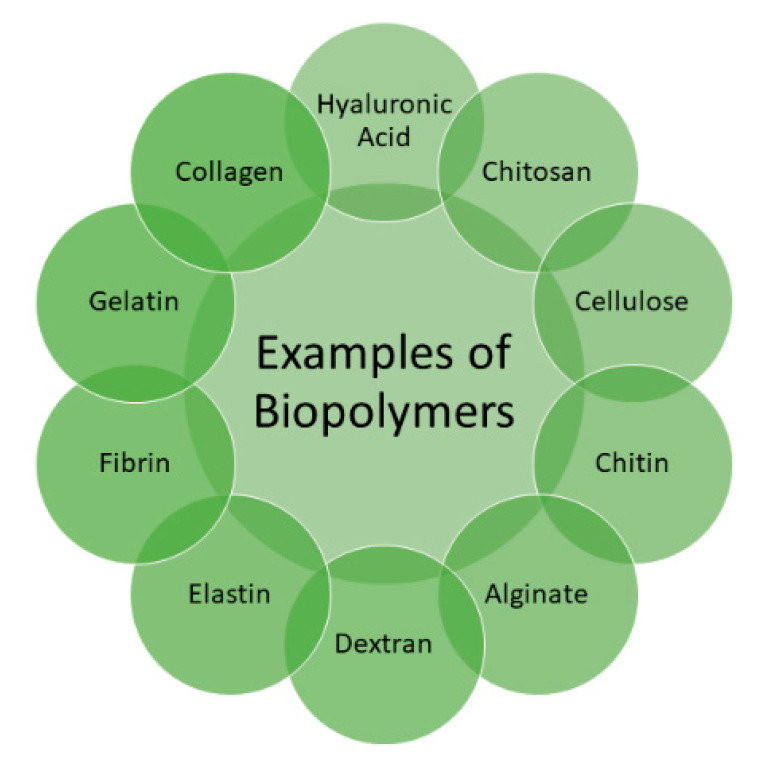
Examples of some biopolymers used in the preparation of wound dressings.

Several biopolymers (Figure 1) are utilized for the preparation of bioactive wound dressings, including hyaluronic acid (HA), cellulose, chitin, chitosan, fibrin, alginate, elastin, dextran, collagen, and gelatin [35], etc. These polymers possess excellent properties that can be significantly beneficial in wound healing, such as non-toxicity, biodegradability, biocompatibility, readily availability, and non-immunogenicity [38]. These polymers can be combined with synthetic polymers to improve their mechanical properties. The factors that must be considered in the preparation of wound dressings include their capability to stop bleeding, combat infections, be easily sterilized, absorb wound exudates, accelerate wound healing and wound debridement, non-toxicity, gas permeability, good water vapor, ease of use, and biodegradability [39]. This review article is based on the therapeutic outcomes of bioactive wound dressings formulated from HA. 

## 2. Classification of Wound Dressing Materials

Wounds, whether acute or chronic, must be properly cared for, and this process involves using wound dressings. These dressings are formulated to be in interaction with the wound environment. Originally, wound dressings were used to make wounds dry for wounds requiring debridement [40]. It is reported that wounds healed quickly in a moist environment, and the dressings must be prepared to absorb exudate and provide moisture [40]. Wound dressings are classified based on their applications into four groups: bioactive dressings, interactive dressings, traditional dressings, and skin substitutes (Table 1) [41]. 

Bioactive wound dressings are responsible for delivering various bioactive agents, including antibacterial agents, growth factors, stem cells, and vitamins, for improving wound healing [42]. These dressings are recognized for their non-toxic nature, biodegradability, and biocompatibility and are commonly formulated from artificial sources or natural tissues such as elastin, chitosan, HA, collagen, and alginate. Some examples of bioactive dressings are hydrogels, sponges, films, nanofibers, wafers, foams, and membranes, etc. [42]. Interactive wound dressings are dressings that act as a barrier against bacterial infection, modify the wound environment’s physiology, improve granulation and re-epithelialization, offer a moist environment for the wound, and enhance water vapor transmission rate (WVTR) with good tensile strength. Biopolymers and synthetic polymers are regularly employed to formulate these dressings. Examples of interactive wound dressings are hydrogels, spray, sponges, foams, films, etc. [38].

Traditional wound dressings, also called passive dressings, are utilized as primary or secondary wound dressings to protect the injury from impurities, stop bleeding, absorb wound exudate, provide a dry environment, and cushion the wound [43]. Due to extreme wound drainage, wound dressing materials become moisturized and usually turn adherent to the injury, making it painful during removal. Examples of passive wound dressings include gauze, bandages, and plaster [43]. Since these dressings fail to offer moisture to the wound environment, they are substituted by modern wound dressing materials with more advanced designs. 

Skin substitutes are formulated to replace damaged skin. They are made up of two tissues, epidermal and dermal layers, obtained from fibroblasts and keratinocytes on a collagen matrix. Skin substitute applications have a short survival time on the wound site and the possibility of infections and disease transmission. Examples of skin substitutes include acellular xenografts, allografts, and autografts [44,45].

## 3. Clinical and Commercially Available Hyaluronic Acid Wound Dressings

**Figure 2:**
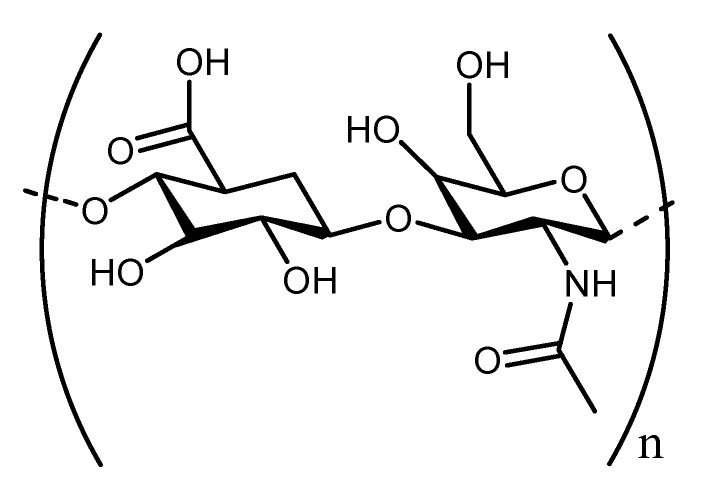
Molecular structure of Hyaluronic Acid.

HA is known as the simplest glycosaminoglycan and it is a constituent of extracellular matrix (ECM) with distinct biological properties, wound healing potential, and physicochemical features. It is composed of disaccharide components consisting of N-acetyl-glucosamine and glucuronic acid (Figure 2) [46,47]. It is found and extracted from rooster combs, synovial fluid, umbilical cord, and vitreous humor [48]. It is biocompatible, non-toxic, biodegradable, exhibits hydrophilic property, non-allergic, and naturally lacks immunogenicity with a broad range of applications [49]. It possesses many essential physiological properties, such as lubrication, space-filling, and structural properties, retention abilities, and ECM water sorption [50]. Excitingly, HA is also recognized as a potential biomaterial for the development of wound dressing. There are various reports that emphasized the wound healing efficacy of HA in stimulating epithelial and mesenchymal cell differentiation and migration, therefore improving collagen deposition and angiogenesis [51].

Several HA-based wound dressings are currently commercially available, such as Hyalosafe^®^, Hyalomatrix^®^, HylaSponge^®^ (Table 2). Hyalosafe^®^ is a transparent film utilized in the wound care and treatment of second-degree burns [52]. The biodegradation of this wound dressing significantly results in the release of HA that promotes epithelial cell proliferation [52]. Hyalomatrix^®^ is a conformable, flexible, and bilayered dermal material that promotes wound closure and dermis regeneration. The bottom layer interacts with the wound, and it is a 3-dimensional fibrous matrix constituent of HYAFF^®^. The top layer is composed of a transparent silicone thin sheet. HYAFF^®^ 11 is an HA-derivative, which is acquired via the esterification reaction of the free HA carboxylic group and benzyl alcohol [52]. The transparency of the top layer is necessary to monitor the wound healing process and the therapeutic outcome of Hyalomatrix^®^ in clinical studies. Clinical studies showed complete wound healing almost a month after treatment. The wounds that were completely closed were found in 85.7% of the patients, whereas 14.3% of them only exhibited a partial re-epithelization [52].

HylaSponge^®^ is a spongy material that has a network system of a huge group of HA molecular chains [53]. This wound dressing possesses the capacity to absorb and release a large volume of water, ensuring skin hydration during the wound healing process. In addition, it serves as an elastic or equilibrium barrier ‘’second skin’’ [53]. It is a gel scaffold and is made up of a mixture of 2.5% sodium hyaluronate and emollients that hinder the dehydration of tissue and support wound healing. There are various types of wounds that can be treated with this gel, including diabetic, leg, and pressure ulcers, and it is suitable for the management of bleeding wounds [54]. Laserskin^®^ is a HYAFF-based material that has a microperforated HA membrane permitting the migration and growth of fibroblasts and keratinocytes on the wound bed. It is regularly utilized for the treatment of acute and chronic wounds [55]. 

Connettivina^®^ is a cream encapsulated with 10 mg/mL of hyaluronate sodium (Na) utilized for the management of skin irritation. It hydrates the wound environment and stimulates migration of cells and skin restoration [56]. Bionect^®^ is a topical formulation composed of 0.2% LMW-HA Na salt and is used to remove the harmful agents, prevent abrasion, and restore skin integrity [57]. Hyalofill^®^ is a colorful cream, non-adherent formulation designed from a formulated-HYAFF. It is used for chronic wound management, including diabetic foot ulcers. This wound dressing is employed as a rope (Hyalofill-R) or a sheet (Hyalofill-F). It absorbs the exudates and produces hydrophilic gel when applied at the wound site [58]. The hydrophilic gel makes a tissue interface rich in HA, providing moisture that stimulates the wound healing mechanism [58]. Although several efforts have been achieved thus far, the clinically and commercially employed HA-based scaffolds used for wound dressings still demonstrate some limitations. Some of the limitations are the possible occurrence of impurities (because of the extraction process employed to get HA), high production costs, low mechanical stability, and limited cell proliferation and adhesion [59]. There are various HA-based scaffolds that are used in wound management (such as hydrogels, sponges, films, foams, membranes) that are currently under development to overcome the shortcomings mentioned above. These wound dressing materials can be loaded with several bioactive agents to improve their therapeutic outcomes.

## 4. Bioactive Agents Loaded Hyaluronic Acid Scaffolds

### 4.1. Hydrogels

**Figure 3:**
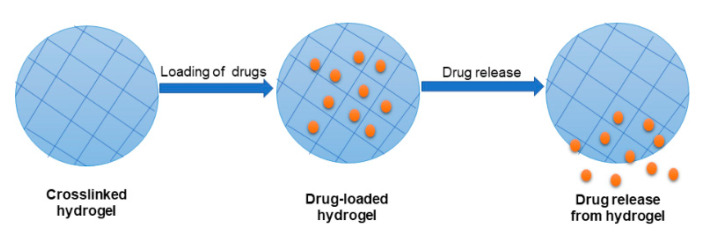
Schematic diagram of hydrogels.

Hydrogels are 3-dimensional polymeric networks that possess an excellent capability to absorb a huge volume of water (Figure 3). It greatly offers a biomimetic and moist environment for the growth of cells. Hydrogels are porous materials that provide the space for nutrients, living cells, gases, and diffusion of waste products [60,61]. These unique features of hydrogels have attracted their application in the field of wound management. Hydrogels are formulated from natural and synthetic polymers, and they exhibit essential properties that can be advantageous in tissue regeneration. HA hydrogels have been broadly evaluated for application in wound healing due to their properties, such as their capacity to offer a moist environment to stimulate cell proliferation and infiltration [62]. Nevertheless, HA-based hydrogels display some limitations, such as rapid degradation and poor mechanical properties [63]. Several strategies are used to overcome these limitations, including crosslinking with synthetic polymers and chemical modification. HA hydrogels can be loaded with various bioactive agents to improve their therapeutic outcomes during wound management and healing.

Hsu et al. formulated gelatin-crosslinked HA-based hydrogels entrapped with recombinant thrombomodulin for wound treatment in diabetic mice [64]. 1-Ethyl-3-(3-dimethylaminopropyl) carbodiimide hydrochloride (EDC) was used as the crosslinking agent that is also known as a broadly-utilized water-soluble crosslinking agent. Fourier-transform infrared spectroscopy (FTIR) results confirmed the successful crosslinking between the polymers. The scanning electron microscopy (SEM) images showed that the hydrogels were porous (around 20–300 µm in diameter). The pore size of hydrogels was decreased as the concentration of HA increased. The in vitro water absorption analysis of hydrogels demonstrated that the water absorption rapidly increased within the first 30 min, and the HA dressings displayed more than 11-fold swelling within one day. HA concentration did not affect the water absorption features of hydrogels. All the HA-based hydrogels exhibited excellent water absorption behavior, which may help absorb wound exudates and promoted good drug absorption at the wound bed. The in vitro drug release kinetics at 33 °C was rapid with over 40% release of thrombomodulin from the HA-based hydrogels in the first 3 h followed by a sustained release. The complete release of thrombomodulin from HA hydrogels occurred in 12 h. The in vivo healing studies on diabetic mice displayed that the hydrogels loaded with thrombomodulin demonstrated significantly more healing effect when compared to plain hydrogels but did not differ from free thrombomodulin solution [64].

Zhang et al. designed dopamine-functionalized HA hydrogels loaded with arginine derivative (antioxidant) for wound healing [65]. FTIR spectra revealed the successful loading of arginine derivative in the hydrogels. The SEM results revealed the morphology of hydrogels, a 3-dimensional microporous network. The size of the pore diameters of the hydrogels became smaller from 14.18 ± 1.54 μm to 9.07 ± 2.17 μm as the concentration of arginine derivative increased. The swelling study showed that all the samples had significant swelling behavior that ranged between 1288.97 ± 325.18% and 4406.05 ± 1233.18, while the water vapor transmission rates ranged between 2004.79 ± 290.56 and 2689.42 ± 783.45 g/m^2^/day. The biocompatibility analysis study of the hydrogels using NIH 3T3 cells revealed that the loading of arginine derivatives in the hydrogels did not have any significant side effects on the cells. The intracellular antioxidant tests using hydrogen peroxide-induced stress damage to the NIH 3T3 cells demonstrated that oxidative stress decreased with increasing concentration of arginine derivative in the hydrogels, which could be advantageous for wound healing. The wound healing experiment in vivo in the rat model displayed that the increase of arginine derivative concentration in hydrogels significantly promotes a faster healing mechanism than the control. Furthermore, the wounds treated with a hydrogel with the highest concentration of arginine derivative showed almost complete healing on day 21 [65].

Liao et al. formulated HA-based hydrogel scaffolds loaded with vancomycin. The in vitro drug release profile demonstrated a slightly faster release of vancomycin from the hydrogels at 37 °C within 72 h [66]. The mean release proportion of loaded antibiotics on the 3rd day was approximately 86%. The gel biodegradation rate was around 20% on the 1st day, 30% on the 7th day, 40% on the 14th day, and complete biodegradation on the 21st day. The antibacterial efficacy of the hydrogel against methicillin-resistant *S. aureus* showed that the average zone of inhibition of vancomycin-loaded hydrogels ranged between 5.7 ± 0.6 and 21.4 ± 0.7 mm post overnight culture. These results can be significantly beneficial in wound treatment [66]. Dong et al. formulated conformable HA-based hydrogels loaded with adipose-derived stem cells for burn injury treatment. The study of the mechanical properties of the hydrogels showed that the storage modulus (G′) of the hydrogels increased significantly at room temperature within 3 min, which specified the gelling points. The gelation happened more quickly with increasing the concentration of the polymer. The in vivo wound healing of the hydrogels on a deep second-degree burn wound murine mice model demonstrated significantly faster closure of the wound and reduced the scar development [67].

Ying et al. formulated in situ collagen-hyaluronic acid hydrogels cultured with fibroblasts (COS-7) and Human microvascular endothelial cells (HMEC) [68]. The swelling analysis of hydrogels demonstrated increased and gradual swelling capacity within 72 h, which reached 45%. This swelling efficiency is beneficial for carrying signal factors and nutrients between cells cultured in the HA hydrogel and cells on the wound, resulting in stimulation of the cell outgrowth. The antibacterial efficacy of hydrogels displayed approximately 47% of *S. aureus* and 55% of *E. coli* were destroyed by post incubating them with the hydrogels at 37 °C for 3 h. COS-7 and HMEC encapsulated within these hydrogels displayed and promoted significant cellular proliferation. The in vivo healing analysis using full-thickness wound in mice showed that wounds treated with the hydrogels displayed a healing process, which was higher compared to the commercial drug and free collagen hydrogel, HA hydrogel samples because the combination of collagen and HA enhanced the wound healing [68]. Wang et al. designed in situ HA hydrogels encapsulated with plasmid DNA encoding vascular endothelial growth factor for the treatment of burn wounds. The in vivo wound healing studies of the hydrogels using Sprague Dawley mice demonstrated accelerated healing on splinted burn wounds, specifically by hindering inflammation reaction and stimulating microvascular development while being biocompatible [69].

Zhao et al. formulated supramolecular HA-based hydrogels encapsulated with epidermal growth factor (EGF) for wound treatment. The physicochemical properties of EGF loaded hydrogels were confirmed by nuclear magnetic resonance (NMR) and UV–Vis spectroscopy. The SEM images revealed that the supramolecular HA-based hydrogels possessed a typical 3D porous morphology. The evaluation of the wound healing mechanism in vivo on a full-thickness skin model revealed a controlled release of EGF from HA hydrogels and excellent healing capacity with respect to angiogenesis, granulation tissue production, and growth factor levels [70]. Makvandi et al. prepared hydrogels based on hyaluronic acid loaded with silver nanoparticles (AgNPs). The rheological evaluation showed that the hydrogels have excellent mechanical properties with gelation temperature near the physiological temperature; hence, they can be simply locally employed on the injured site. Cytotoxicity assay displayed that the HA hydrogels have excellent biocompatibility on L929 cells. The antibacterial efficacy of hydrogels loaded with Ag NPs exhibited a remarkable growth inhibitory effect against B. subtilis and *E. coli*. The in vitro model of wound healing demonstrated that the AgNPs nanoparticle-loaded hydrogels permit accelerated wound closure and restoration than the control [71].

Larrañeta et al. formulated hyaluronic acid hydrogel crosslinked with Gantrez S97 and loaded with methylene blue (used as model biomolecule) employing a solvent-free method for biomedical applications. The crosslinking was confirmed by IR and Dynamic Scanning Calorimetry (DSC) analysis. The in vitro drug release profile of HA hydrogels showed sustained methylene blue release over 48 h. The swelling analysis demonstrated a high swelling efficiency of hydrogels in water and phosphate buffer saline solution. The in vitro antibacterial activity exhibited important reductions of approximately 98.2% and 98.4% of P. mirabilis and *S. aureus* after 4 h of incubation. These results revealed that HA hydrogels could be beneficial in wound treatment [72]. Nejad et al. designed double crosslinked HA-based hydrogels encapsulated with dexamethasone and poly(l-lactide-co-glycolide) (PLGA) nanoparticles for wound treatment. The physicochemical properties of hydrogels were confirmed by NMR, FTIR, and gel permeation chromatography (GPC). The in vitro drug release profiles of the hydrogels showed no burst release of the nanoparticles in the first hours. However, after 1 day, they displayed 4.8% and 8.3% release. The cytotoxicity study using MTT assay on the hydrogels demonstrated high cell viability of HFFF2 human fibroblast cells. These results also revealed that HA hydrogels are suitable for wound care [73].

Da Silva et al. designed HA-based spongy hydrogels loaded with human adipose stem cells for diabetic wound treatment. These spongy hydrogels demonstrated improved diabetic wound healing by significantly modulating the inflammatory response and re-epithelialization to stimulate effective neoinnervation [74]. Rao et al. formulated hyaluronan hydrogels loaded with ZnO nanogel-like structures for application in wound dressing. The physicochemical properties of hydrogels were successfully confirmed by 1H NMR, FTIR, and X-ray diffraction (XRD). The SEM pictures without ZnO nanoparticles displayed interconnected porous networks. Furthermore, the encapsulation of the ZnO nanoparticles significantly promoted a porous structure. The swelling study of hydrogels showed a higher swelling capacity. A hemostatic examination of the HA hydrogels demonstrated excellent hemostatic properties of ZnO-loaded HA hydrogels. In vitro antimicrobial study of the hydrogels against *E. coli* and *S. aureus* strains revealed good antibacterial activity of the hydrogels [75]. These HA hydrogels are promising scaffolds for cell adhesive with hemostatic properties and antimicrobial materials for potential application in wound treatment.

### 4.2. Films/Membranes

Films are flexible, transparent, and elastic materials made up of polymers-they permit oxygen and carbon dioxide exchange and diffusion of water vapor from the injury site and prevent penetration of bacterial/other microbial pathogens [76]. The transparency of films allows the monitoring of the wound continuously, without removing it [76]. These dressings also stimulate the autolytic eschar debridement. However, films demonstrate a reduced capacity to absorb the wound exudate, making them unsuitable for high exuding wounds and may cause trauma if not removed appropriately [77]. Currently, HA films are developed by incorporating bioactive molecules (such as natural product extract, growth factors, inorganic compounds, and antibiotics) to improve their biological application. Also, HA-based are functionalized with HA-derivatives or other polymers [78].

Duan et al. formulated HA-based films incorporated with curcumin for wound healing [79]. These films were characterized by NMR, DSC, and FTIR to confirm their expected physiochemical properties. The swelling analysis showed that curcumin-loaded films and plain films swelled rapidly within 15 min and reached a swelling equilibrium at 2 h, demonstrating that all the hydrogels have outstanding water absorption. The equilibrium swelling ratio ranged between 879% and 919%. The in vitro skin irritation examination revealed that curcumin-loaded films did not cause skin irritation. The cytotoxicity study of free curcumin and curcumin grafted films on the L929 cells using MTT assay showed no adverse effect within 72 h. The cell viability value was above 90%, demonstrating that the films had no significant inhibition effect on the proliferation of L929 within a selected concentration. The in vitro antibacterial analysis on *S. aureus* and *E. coli* of the films showed an inhibitory concentration of 0.15 g/mL for *S. aureus* and 0.20 g/mL for *E. coli*. The hemolysis test demonstrated no significant hemolysis with concentrations of films reaching up to 15 mg/mL, revealing that the curcumin-loaded HA films have excellent biocompatibility. The wound healing test showed that the wound size of each group decreased as time extended. On the third day, all the groups demonstrated a constriction on the wound site [79].

Abou-Okeil et al. prepared sodium-alginate crosslinked HA films loaded with AgNPs and sulfadiazine as topical bioactive wound dressings [80]. The SEM images exhibited crosslinked films that were homogeneous with no phase cracks. The drug release kinetics in vitro showed that the release of sulfadiazine was higher at pH 4.1 compared to pH 7.2, revealing a diffusion of sulfadiazine from a swelled film structure at pH 4.1 compared to the compact structure of the film at pH 7.2. The in vitro antibacterial analysis showed that a combination of sulfadiazine (as an antibiotic) with silver nanoparticles in the films results in more enhanced antimicrobial properties. The wound healing study in vivo using wounded mice showed that after three weeks, all the wounds treated with dual-loaded HA-based films were completely closed compared to the control group [80].

Abednejad et al. formulated HA-based films embedded with functionalized Zeolite Imidazolate Frameworks (ZIF-8), which can provide good mechanical and antibacterial properties [81]. DLS analysis of ZIF-8 displayed a uniform distribution of particles with a mean particle size of approximately 44 nm, confirming their nanosize. FTIR and XRD spectrums confirmed successful encapsulation of ZIF-8 in HA films, while SEM images demonstrated appropriate dispersion of frameworks in HA material. Analysis of the mechanical properties of the films showed Young’s modulus of 176 K Pa and decreased water contact angle of 27.7, showing improvement in hydrophilicity. The swelling results showed the swelling time was 90 min for HA films with a maximum water uptake of about 305 ± 5%, whereas the swelling time was 250 min as the percentage of ZIF-8 reached 2% by weight. The cytotoxicity test of plain films and ZIF-8 on L929 cells showed a high cell viability of more than 98%. The antibacterial results from the disc diffusion method confirmed better growth inhibition for the films loaded with ZIF-8 compared to the free films against *E. coli* and *S. aureus*. These results demonstrated that HA-based films loaded with ZIF-8 are beneficial for treating wounds infected with microbial pathogens [81].

Contardi et al. prepared transparent bilayered films based on HA and Polyvinylpyrrolidone (PVP). It was encapsulated with antiseptic (neomercurocromo) and ciprofloxacin for wound dressing [82]. The morphological analysis of the bilayered films using SEM showed that the top layer is approximately 60 µm thick and the bottom layer is thicker, being near 200 µm. The FTIR results displayed the expected functional groups of the loaded ciprofloxacin and antiseptic in the HA films. The in vitro drug release at physiological conditions demonstrated a drug release reaching almost 100% within the initial 24 h. The antibacterial results showed that bilayered films formed a zone of inhibition of 30.6 (±1.9), 42.9 (±3.2), 39.5 (±2.0) mm against *S. aureus*, *P. aeruginosa*, and *E. coli*, respectively, revealing their good antibacterial activity. The in vivo biocompatibility and bioresorption studies of the films in a full-thickness wound on mice model showed no observable side effects. The bilayered films displayed a slow, consistent tendency in exudate absorption and film biodegradation. After 2 h, the films started to absorb a large amount of exudates [82].

Michalska-Sionkowska et al. designed thin films that are based on HA, chitosan, and collagen. The films were loaded with gentamicin. The contact angle analysis showed that the hydrophilicity of film decreased when gentamicin was added. The water vapor transmission analysis showed that the presence of gentamicin in thin films resulted in increased water vapor permeability. The in vitro antimicrobial results revealed that the films based on HA and other biopolymers encapsulated with gentamicin inhibited the growth of *P. aeruginosa*, *S. aureus*, and *E. coli*. In contrast, films without gentamicin displayed no antibacterial activity on these bacterial strains [83].

Tamer et al. formulated and evaluated polymeric membranes prepared from HA and chitosan. They were loaded with glutathione for enhanced wound treatment [84]. The successful preparation and incorporation of glutathione in the HA-based membranes were confirmed by FTIR, while their thermal stability was assessed by TGA analysis. The water uptake study showed that the encapsulation of glutathione into the membranes induced a small rise in water uptake from 230.3 ± 8.3% to 240.8 ± 7.9% that is attributed to the hydrophilic property of glutathione. The porosity analysis utilizing ethanol as a hydrophilic solvent demonstrated that the addition of glutathione increased the porosity of plain membrane from 37.53 ± 1.88% to 43.26 ± 2.16%, which is useful for promoting their surface area and the adsorption capacity. The wound healing results in vivo employing a rat model demonstrated rapid wound healing for the drug-loaded membranes. Furthermore, in vivo study results revealed that the membranes loaded with glutathione are an appropriate wound dressing for treating chronic wounds [84].

Abednejad et al. designed polyvinylidene fluoride–hyaluronic acid membranes loaded with active pharmaceutical ingredient ionic liquids (API-ILs) for wound healing. The SEM analysis showed that the increase in the concentration of polyvinylidene fluoride resulted in spongy region development, which can contribute to more polymeric chains and higher viscosity. Finger-like materials make the membrane able to load API-ILs, while the spongy region was significant in API-ILs release, as it controls the passage of the therapeutic agent via the pores. The uptake analysis revealed that the shape, size, and interconnectivity of pores affected the membrane uptake of API-ILs. Cell adhesion of fibroblasts on HA-based membrane surfaces and cell viability evaluation confirmed enhanced viability and adhesion of fibroblasts on the membranes. Healing assay evaluated with fibroblasts demonstrated that the bilayer membranes containing API-ILs are not detrimental to wound healing [85].

Abid et al. formulated collagen combined HA-based membranes loaded with hydroxylapatite + β-TCP to restore surgical bone defects. The in vivo results using rabbits revealed that the loading of the hydroxylapatite + β-TCP in HA membranes was found to progress into the best progressive phases of the bone repair process at the second and fourth weeks [86]. Figueira formulated polycaprolactone-HA electrospun bilayer nanofibrous membrane encapsulated with salicylic acid for tissue regeneration [87]. The physicochemical properties of the nanofibrous bilayer membrane loaded with salicylic acid were evaluated, and the achieved results demonstrated that the formulated electrospun scaffolds exhibited suitable mechanical properties, ideal porosity, controlled water loss, and a significant salicylic acid drug release mechanism. The in vitro cytotoxicity studies demonstrated that HA-based membranes did not show any side effects on human fibroblast cells since the cells were able to proliferate, migrate, and adhere to the surface of the membranes. Furthermore, no biofilm development was observed on the surface of the HA membrane. The antimicrobial analysis showed higher growth inhibition against *S. aureus*. The results reveal that these membranes displayed suitable properties to be employed in wound treatment [87].

### 4.3. Sponges

Sponges are wound dressings that can absorb large quantities of wound exudates, and they provide a moist environment for the wound because of their high porosity, swelling profile, and biodegradability [88]. Generally, sponges are non-adhesive and require secondary wound dressing or bandages/tapes to keep them at the wound site [63]. HA derivatives or other polymers have been used in combination with HA to overcome the poor mechanical properties demonstrated by HA-based sponges. Mohandas et al. prepared fibrin nanoparticles encapsulated with vascular endothelial growth factor (VEGF) and loaded them in formulated chitosan-HA composite sponge for diabetic wound dressing [7]. SEM images of VEGF loaded fibrin nanoparticles displayed spherical shape with a size range between 150 and 180 nm, while DLS analysis exhibited a mean particle size of 180 nm with a polydispersity index (PDI) of 0.4 and an average negative surface charge of 28 mV. The FTIR spectrum showed the expected functional groups of biopolymers and confirmed the successful encapsulation of nanoparticles in the sponges. The porosity experiment demonstrated that all the sponges possessed a porosity that ranges between 65–75%. The mechanical analysis results displayed an elongation at break, which showed the flexibility of HA-based sponges that ranged between 10–20%, while the tensile strength ranged between 0.15 and 0.02 MPa. The flexibility of the scaffold was improved by increasing HA concentration showing that the sponges can be placed on any tissues without breaking [7]. The swelling examination was performed for 7 days, and the swelling ratio ranged between 8 and 12% for all the sponges. The encapsulation of VEGF did not result in any significant difference in swelling. The in vitro release profile demonstrated an initial burst release of 29% VEFG from the sponges at the first 2 h, followed by a 64% release of VEFG at 3 days which was sustained for 7 days at 37 °C. The initial burst release can speed up angiogenesis to accelerate the wound healing process. The cytotoxicity studies of sponges in HDF cells revealed more than 85% cell viability. Furthermore, endothelial cells loaded on the sponges were well proliferated and demonstrated capillary-like tube development, which is a significant process in wound healing angiogenesis. These scaffolds offer a promising approach for the management of diabetic wounds [7].

Fiorica et al. prepared α-elastin crosslinked HA sponges incorporated with VEGF for wound healing. The swelling analysis showed a swelling percentage of 217 ± 11%, thus revealing a high efficiency of the sponges to absorb wound exudates. The biodegradation analysis of the sponges showed that the sponges degraded in the presence and absence of hyaluronidase (HAase). The cytocompatibility analysis of sponges showed that the VEGF released from the sponges was active in promoting the proliferation of HUVEC cells, and the cell viability was not affected by the presence of VEGF in the plain sponges [36]. Ross et al. formulated sponge-like dressings from chitosan glutamate and HA. They were loaded with platelet lysate for the treatment of chronic wounds. The results from this study revealed that platelet lysate stimulates keratinocyte epithelialization and controls fibroblast matrix deposition, offering a molecular basis for the capability of such hemoderivative to heal chronic wounds. Furthermore, platelet lysate acts on the wound healing process by accelerating the formation of new tissues [89].

Lu et al. reported chitosan-l-glutamic acid-HA sponges loaded with Ag NPs as wound dressings [90]. FTIR and XRD spectrums confirmed the successful preparation of nanoparticle-loaded sponges. The porosity analysis of the sponges demonstrated that the loading of the nanoparticles significantly increased the porosity of sponges from 83.5% to porosity that ranges between 90.3% and 94.1%. The mechanical properties analysis revealed Young’s moduli of the Ag nanoparticle loaded sponges (0.2 ± 0.01 MPa), demonstrating that these two scaffolds could resist distortion and had excellent mechanical strength. The antimicrobial analysis of sponges showed that the addition of Ag nanoparticles increased the size of the inhibition zone in a concentration-dependent mode against *E. coli* and *S. aureus*. The in vitro cytotoxicity examinations of the sponges using MTT assay demonstrated cell viability that was more than 80% on L929 cells. The in vivo healing analysis of sponges on full-thickness skin wounds using rabbits revealed healing on the third day, with slight inflammation and approximately 47% wound closure for the plain sponges, 69% for the sponges loaded with Ag nanoparticles, and only 5% wound contraction for the control (gauze) [90].

Anisha et al. prepared antimicrobial chitosan-HA sponges loaded with nanosilver for wound healing of infected diabetic foot ulcers. The porosity analysis of nanosilver encapsulated HA-based sponges displayed a porosity of about 73%, whereby the control sponges exhibited a porosity of 63%. This enhanced porosity influenced the increased water uptake of the sponges. The swelling evaluation indicated higher swelling and water uptake capacity for the nanoparticle silver incorporated sponges. The in vitro antimicrobial analysis demonstrated that nanosilver incorporated sponges effectively reduced the growth of *E. coli*, *S. aureus*, MRSA, K. pneumoniae, and *P. aeruginosa*. These sponges are potential wound dressings suitable for managing diabetic foot ulcers infected with antimicrobial-resistant bacteria [91]. Anisha and co-workers designed chitosan–HA composite sponge loaded with chondroitin sulfate nanoparticles. SEM and DLS analysis of the nanoparticles demonstrated a particle size that ranges between 100 and 150 nm. HA-based sponges exhibited a porosity of 67% and displayed improved blood clotting ability and swelling capacity. The cytotoxicity evaluations of the sponges displayed more than 90% cell viability on human dermal fibroblast (HDF) cells. These scaffolds also exhibited improved proliferation of HDF cells within 48 h [16].

### 4.4. Nanofibers

Nanofibers are promising materials suitable for rapid and complete wound healing of chronic wounds, including burns, providing significantly enhanced outcomes more than the conventional wound dressings. There are several distinct properties of nanofibers, such as nanoscale structure, porosity, and large surface area. These features play vital roles in cell proliferation and attachment [21]. Electrospinning can be employed to prepare nanofibers using biopolymers or synthetic polymers. It is the most broadly promising and widely studied method for the preparation of nanofibers [2]. Nanofibers designed by electrospinning have triggered great interest in different applications such as drug-delivery systems, wound-dressing materials, and bio-nanotextiles because of their large surface-area-to-volume proportion [92,93]. 

El-Aassar et al. prepared and evaluated PVA crosslinked hyaluronic acid-based nanofibers embedded with silver nanoparticles via electrospinning technique for wound healing [37]. The physicochemical properties and successful embedding of nanoparticles in the nanofibers were confirmed by UV-vis spectroscopy, FTIR, and Raman spectroscopy. The SEM analysis of nanofibers demonstrated an approximate average size diameter between 244 nm and 326 nm. The TEM showed Ag NPs homogeneous distributed within the HA-based nanofibers. The SEM and TEM results confirmed the electrospinning process of nanofibers embedded silver nanoparticles. The mechanical properties analysis showed the tensile stress of the silver nanoparticle-loaded nanofibers (~4.1 MPa), which was almost two times stronger than the plain nanofibers (~1.9 MPa). The antibacterial assessments showed that nanofibers loaded with silver nanoparticles possessed high efficacy against *B. subtilis*, *E. coli*, and *E. coli* compared to the plain nanofibers [37].

The wound healing studies of nanofibers revealed the absence of abscess development or hypertrophic scars. These results showed the wound contracting capability of the silver nanoparticle-loaded nanofibers and free nanofiber treated groups. The nanofibers induced wound healing from the 8th day, and the epithelization time was on day 14. In contrast, the Garamycin^®^ cream and control group epithelization occurred after more than 2 weeks. The nanoparticle embedded nanofibers and the plain nanofibers treated groups displayed important wound healing efficacy compared to other groups treated with Garamycin^®^ cream and control group excision wound model [37].

### 4.5. Other HA Scaffolds

There are HA-based scaffolds that can be used in wound healing as potential bioactive dressings such as bandages, topical gels, microspheres, nanoparticles, hydrocolloids, foams, transdermal/matrix patches, etc. [94]. Hussain et al. reported HA-functionalized nanoparticles for the co-delivery of resveratrol and curcumin for chronic diabetic wound treatment [95]. Dynamic Laser Light Scattering (DLS) analysis of the HA nanoparticles demonstrated a mean particle size of about 200 nm, a surface charge of more than ±30 mV, entrapment capacity of ~90% of curcumin, and resveratrol. FTIR and XRD analysis showed the successful preparation of the co-loaded nanoparticles. The in vitro drug release studies simulating the conditions of diabetic wounds’ microenvironment (pH ranges between 6.2 and 8.5) were performed. The release analysis at pH 7.4 and 37 °C revealed the release of both drugs loaded followed biphasic release mode where a first burst release of both drugs occurred in the first 8 h followed by slower and sustained drug release. These results and properties confirmed that these nanoparticles could be very suitable for chronic diabetic wound treatment [95].

Abdel-Mohsen and co-workers formulated silver nanoparticle-loaded HA-based fabrics for chronic wound management. The resultant fabrics loaded with nanoparticles displayed consistent dispersion throughout the HA-based fabrics from SEM analysis, TEM and DLS analysis, demonstrating an outstanding distribution of silver nanoparticles with 25 ± 2 nm. The XRD results showed that the silver nanoparticles increased the crystallinity of the formulated fabrics as well as the thermal stability. The in vitro antimicrobial analysis of the fabrics against *E. coli* K12 was significant. The cytotoxicity studies showed that the fabrics did not reveal any cytotoxic effect against the human keratinocyte cell line (HaCaT). In vivo wound healing analysis using non-diabetic/diabetic rat models showed that the formulated fabrics loaded with silver nanoparticles displayed strong healing efficacy compared to the free HA fabrics and significantly accelerated the wound healing process [96].

Eskandarinia et al. formulated polyurethane-HA nanofibrous wound dressings incorporated with propolis. The FTIR spectrums showed the expected functional groups from the polymers and confirmed the successful incorporation of propolis. The analysis of mechanical properties of these wound dressings showed the tensile strength ranging between 4.91 ± 0.5 and 3.07 ± 1.1 MPa while the elongation at break ranged between 379.8 ± 23 and 453.6 ± 38.5 MPa. The mechanical properties showed that these nanofibrous scaffolds are appropriate for wound dressing. The antimicrobial analysis showed that propolis-incorporated nanofibrous scaffolds caused significant inhibition zones against *E. coli* and *S. aureus*. The wound healing study in vivo using Wistar rats demonstrated a significantly accelerated healing process compared to the control. The wounds treated with the propolis loaded nanofibrous scaffolds closed on day 14, whereas the wounds treated with the free polyurethane and control groups were still open [97].

Fahmy and co-workers formulated chitosan-HA non-woven fabric wound dressing incorporated with silver nanoparticles. The TEM analysis revealed the mean particle size of the AgNPs was less than 13 nm. The elemental analysis of the dressings loaded with nanoparticles confirmed the presence of Ag NPs in the non-woven fabric wound dressing. The thermogravimetric analysis confirmed the higher thermal stability of the formulated chitosan-HA non-woven fabric wound dressings [98]. Guzińska et al. formulated Antibacterial HA-based foams incorporated with zinc oxide nanoparticles. The in vitro antimicrobial analysis showed a significant reduction of the number of *E. coli* bacteria after an hour for the nanoparticles loaded foams compared to the plain foams. The foams loaded with 3% cephalosporin revealed only half of the same reduction rate. These results demonstrated that these materials are suitable for the treatment of bacterial-infected wounds [99].

Gokce et al. formulated a 3-dimensional dermal matrix based on HA loaded with resveratrol for diabetic wound treatment [100]. The SEM images demonstrated that the pore size of the dermal matrix was approximately 100 µm. The water uptake analysis showed the water uptake capacity of more than 80% arising from the porous material of the dermal matrix, resulting in good absorbent characteristics. Other evaluations of the mechanical properties of the dermal matrix were as follows: 18.3 ± 0.3 N mm for compressibility, 21.2 ± 0.5 N for hardness, 0.96 ± 0.1 for elasticity, and 01 ± 0.01 for cohesiveness. The sterility analysis showed that these formulations are sterile. The in vivo wound healing analysis of the 3-dimensional dermal matrix loaded with resveratrol on wounded diabetic rats showed that the wounds were fully healed at the end of 2 weeks compared to those treated with resveratrol solution and the plain dermal matrix [100]. Dias et al. formulated the HA-based matrix loaded with anti-inflammatory jucá extract. The cytotoxicity analysis of the extract-loaded matrix showed good cytocompatibility with RAW 264.7 macrophages with cell viability of more than 85% after 24 h. The dressings offered a broad range of oxygen permeability and water vapor ((2.9–14.7) × 1014 kg/(s m Pa)), which make them potentially appropriate for the treatment of different wound types at various wound healing phases [101]. All the HA-based scaffolds (hydrogels, films, membranes, sponges, nanofiber, and others) discussed above are summarized in Table 3 below:

## 5. Conclusions

This review article discussed HA-based scaffolds as potential bioactive wound dressings. There are several features that are displayed by these scaffolds, including good biocompatibility on various types of human cells, the ability to be chemically modified, non-toxicity, easy methods of preparation—making them affordable with enhanced biodegradability, hydrophilicity, and their ability to be loaded with bioactive agents. The advantages that make HA-based scaffolds potentially suitable for the management of wounds include high swelling capacity, good porosity, enhanced water vapor transmission rate, good water absorption, antibacterial properties, and excellent mechanical properties such as young modulus, flexibility, elasticity, and stability. Although the encapsulation of various bioactive agents may affect the aforementioned advantages, it can also result in an accelerated wound healing process by triggering several biological processes (such as fibrin clot, fabrication, and release of pro-inflammatory cytokines and interleukins, and keratinocytes/fibroblasts proliferation). Furthermore, the loading of antibacterial agents in HA-scaffolds results in enhanced protection of the wounds from bacteria invasion and treats bacteria-infected chronic wounds. Based on the reports of several researchers, there is no doubt that HA-based wound dressings are promising therapeutics for treating chronic wounds.

## 6. Future Perspective

Utilizing HA for the design of wound dressings has increased significantly in recent years with promising therapeutic outcomes. However, in the design of wound dressings, specific factors are usually considered depending on the type of wounds. HA is one of the significant components of the extracellular matrix of the human skin and is very important in inducing the release of proinflammatory cytokines and enhancing fibrin clot formation.

Furthermore, HA’s unique features make it a promising material for biomedical applications such as wound dressings, drug delivery systems, etc. HA-based wound dressings have displayed enhanced mechanical properties, good porosity, promoted oxygen and oxygen and nutrients exchange, absorbed a significant amount of wound exudate, induced cell proliferation, and improved re-epithelization and granulation, revealing skin regeneration capability. HA has been crosslinked with other polymers resulting in enhanced mechanical properties. The loading of bioactive agents into the scaffolds resulted in good antibacterial activity, which is appropriate for treating infected wounds. Some of the bioactive agents loaded in HA-based wound dressings promoted skin regeneration, rapid wound closure, and the absence of scar tissues. 

In the future, there is a need for more studies on the efficacy of loading two or more bioactive agents and growth factors in HA-based wound dressings for skin regeneration. Designing HA-based scaffolds via new techniques with excellent mechanical properties, outstanding biological outcomes, and affordable production costs without using toxic materials also needs to be investigated. The in vitro and in vivo studies of HA-based scaffolds reported by several researchers demonstrate that HA is a promising material and these scaffolds with outstanding therapeutic effects have the potential to reach clinical use in the near future.

## Figures and Tables

**Table 1:** Summary of classification of wound dressing materials.

Types of Wound dressings	Examples	Functions in Wound Management	References
Bioactive dressings	hydrogels, wafers, sponges, films, nanofibers, foams, and membranes	They are responsible for delivering bioactive agents such as antibiotics, stem cells, growth factors, and vitamins to improve the healing process.	[35]
Interactive dressings	hydrogels, spray, sponges, foams, and films	They act as a barrier against bacterial infection, modify the physiology of the wound environment, improve granulation and re-epithelialization, offer a moist environment for the wound, and enhance WVTR with good tensile strength	[31]
Traditional/passive dressings	gauze, bandages, and plaster	They protect the wound from impurities, stop bleeding, absorb wound exudate, and provide cushion to the wound	[36]
Skin substitutes	xenografts, allograft, and autografts	They replace the damaged skin	[37,38]

**Table 2:** Commercially available HA-based scaffolds for wound dressing and their specific functions.

Commercially Available HA-Based Wound Dressings	Forms of Wound Dressings	Functions in Wound Healing Application and Wound Types	Reference
Hyalosafe^®^	Film	It is transparent and allows the wound healing process to be easily monitored. It is suitable for the treatment of moderate exuding wounds and surgery wounds.	[45]
Hyalomatrix^®^	Membrane	It promotes skin re-epithelialization. It is used for the treatment of full-thickness wounds, second-degree burns, venous ulcers, pressure ulcers, and chronic vascular ulcers,	[45]
HylaSponge^®^	Sponge	It absorbs a large volume of water and hydrates the skin to promote wound healing development. It is used to treat acute and chronic wounds.	[46]
Hylase Wound Gel^®^	Gel	It prevents tissue dehydration, which in turn promotes the wound healing process. It is suitable for wound care of pressure, leg and diabetic ulcers, and bleeding wounds.	[47]
Laserskin^®^	Scaffold	It promotes the migration and growth of autologous fibroblasts and keratinocytes to the wound bed. It is appropriate for acute and chronic wounds.	[48]
Connettivina^®^	Cream	It provides a hydrated environment that promotes skin regeneration. It is used to treat skin irritations.	[49]
Bionect^®^	Topical Solution	It is used to avoid abrasion and for the removal of harmful foreign agents. It is also used to treat skin irritations.	[50]
Hyalofill^®^	Cream	The hydrophilic gel created on the wound by this cream and wound exudates is rich in the HA tissue interface, offering moisture to promote the wound healing process. It is used for the treatment of chronic wounds, including diabetic foot ulcers.	[51]

**Table 3:** HA-based Bioactive Wound dressing.

HA-Based Wound Dressing	Polymer Used for Cross-Linking	Loaded Bioactive Agent	Therapeutic Outcomes	References
Hydrogels	Gelatin	Recombinant thrombomodulin	High swelling capacity, sustain drug release mode, and good diabetic wound healing effect.	[64]
	None	arginine derivative	Non-toxic and accelerated wound healing process.	[65]
	None	vancomycin	Average zone of inhibition against methicillin-resistant *S. aureus.*	[66]
	None	adipose-derived stem cell	Faster wound closure on deep second-degree burn wound reduced scar formation.	[67]
	Collagen	COS-7 and HMEC cells	Average antibacterial efficacy against *S. aureus* and *E. coli,* and accelerated healing process on full thickness-wound.	[68]
	None	Plasmid DNA encoding VEGF.	Accelerated healing on burn wounds.	[69]
	None	EGF	Superior wound healing results in a full-thickness skin wound model.	[70]
	None	Ag NPs	Excellent biocompatibility on L929 cells and high growth inhibitory effect against *E. coli* and *B. subtilis.*	[71]
	Gantrez S97	methylene blue	Sustained drug release kinetics over 2 days and good bactericidal effect against *S. aureus* and *P. mirabilias.*	[72]
	PLGA	Dexamethasone and PLGA nanoparticles	Slow drug release and good cell viability on HFFF2 human fibroblast cells.	[73]
	None	human adipose stem cells	Improved diabetic wound healing.	[74]
	None	ZnO nanogel-like structures	Higher swelling capacity, good hemostatic properties, and outstanding antibacterial efficacy against *S. aureus* and *E. coli*	[75]
Films	none	curcumin	Excellent cell viability on the L929 cells and good wound closure effect.	[79]
	Na-alginate	Ag NPs and sulfadiazine	Synergistic antibacterial activity and good wound healing process.	[80]
	None	ZIF-8	Good mechanical properties, high cell viability, and better growth inhibition against *E. coli* and *S. aureus.*	[81]
	PVP	Neomercurocromo and ciprofloxacin	Good bactericidal efficacy and with no significant side effects in vivo.	[82]
	chitosan and collagen	gentamicin	Higher growth inhibition against growth of *P. aeruginosa*, *E. coli,* and *S. aureus.*	[83]
Membranes	chitosan	glutathione	High water uptake and faster wound healing mechanism.	[84]
	Polyvinylidene fluoride	API-ILs	Enhanced cell viability and adhesion of fibroblasts on membranes.	[85]
	collagen	hydroxylapatite + β-TCP	Advanced stages of the bone repair process.	[86]
	Polycaprolactone	salicylic acid	High antibacterial activity and good cell viability.	[87]
Sponges	None	VEGF	Sustained drug release.	[7]
	α-elastin	VEGF	Good cell viability.	[36]
	chitosan glutamate	platelet lysate	Good healing process.	[89]
	chitosan-l-glutamic acid	Ag NPs	Excellent mechanical properties and high antibacterial activity against *S. aureus* and *E. coli*.	[90]
	Chitosan	Nanosilver	High swelling capacity and water uptake ability and reduced growth of *E. coli*, *S. aureus*, MRSA, *K. pneumoniae,* and *P. aeruginosa.*	[91]
	chitosan	chondroitin sulfate nanoparticles	High cell viability on HDF cells and improved cell proliferation within 2 days.	[16]
Nanofibers	PVA	Ag NPs	Good mechanical properties and high antibacterial efficacy against *E. coli, B. subtilis,* and *S. aureus.*	[37]
nanoparticles	None	resveratrol and curcumin	Sustained drug release.	[95]
Fabrics	None	Ag NPs	Good bactericidal activity against *E. coli K12* and accelerated healing process.	[96]
Nanofabrious scaffolds	polyurethane	Propolis	Good mechanical properties and significant inhibition zones against *S. aureus* and *E. coli*.	[97]
fabric wound dressing	Chitosan	Ag NPs	Higher thermal stability.	[98]
foams	None	zinc oxide nanoparticles	Good antibacterial efficacy.	[99]
dermal matrix	None	resveratrol	High water uptake capacity, good mechanical properties, and accelerated wound healing at the end of 2 weeks.	[100]
matrix	None	jucá extract	High cell viability, high water vapor, and oxygen permeation.	[101]
